# Editorial: Molecular and cellular mechanisms involved in inflammation, metabolism and oxidative stress induced by coronaviruses

**DOI:** 10.3389/fmicb.2023.1182531

**Published:** 2023-03-22

**Authors:** Sandro Massao Hirabara, Renata Gorjao, Gabriel Nasri Marzuca-Nassr, Kaio Fernando Vitzel, Marco Aurélio Ramirez Vinolo, Laureane Nunes Masi

**Affiliations:** ^1^Interdisciplinary Post-Graduate Program in Health Sciences, Cruzeiro do Sul University, São Paulo, Brazil; ^2^Departamento de Ciencias de la Rehabilitación, Universidad de La Frontera, Temuco, Chile; ^3^School of Health Sciences, College of Health, Massey University, Auckland, New Zealand; ^4^Laboratory of Immunoinflammation, Department of Genetics, Evolution, Microbiology, and Immunology, Institute of Biology, University of Campinas, Campinas, Brazil

**Keywords:** SARS-CoV-2, COVID-19, metabolic pathways, chronic diseases, prevention and treatment

The Coronavirus Disease 2019 (COVID-19) pandemic emerged at the end of 2019, imposing several challenges worldwide. A prompt response of the scientific community was essential for understanding the pathophysiological mechanisms involved on the infection caused by the Severe Acute Respiratory Syndrome Coronavirus 2 (SARS-CoV-2) and its variants, as well as to develop interventions to prevent or attenuate the severe outcomes of this disease (Triggle et al., 2020; Harvey et al., 2021; Hirabara et al., 2022; Singh et al., 2022; Hillary and Ceasar, 2023). Aggravation of the illness condition induced by the SARS-CoV-2 infection has been associated with exacerbated inflammation, metabolism alteration, and elevated oxidative stress. The understanding of the molecular and cellular mechanisms involved in the development of these processes during COVID-19 is fundamental for directing and supporting effective treatments and strategies to combat the disease.

Several groups have previously demonstrated that SARS-CoV-2 modulates inflammation, metabolism, and oxidative stress in different cells and tissues. The present Research Topic received studies related to the comprehension of the molecular and cellular mechanisms involved in the development of the inflammatory process, metabolism modulation, and oxidative stress in response to the SARS-CoV-2 infection. Particularly, those addressing the molecular mechanisms in different tissues and experimental models, as well as indicating potential interventions were welcomed. The main topics of this collection included: (a) molecular and cellular mechanisms of inflammation, metabolism, and oxidative stress induced by SARS-CoV-2; (b) crosstalk among these processes during the coronavirus infection; (c) effect of aging and comorbidities on inflammatory process, metabolism modulation, and oxidative stress; (d) characterization of molecular targets associated with these processes; (e) potential strategies for the prevention or treatment of the inflammatory response, metabolism deregulation, and oxidative stress process based on molecular targets of signaling pathways; and (f) identification of the mechanisms involved by different nutritional compounds or dietary regimens in the modulation of inflammation, metabolism, and oxidative stress.

The interplay among the compositional changes in the gastrointestinal microbiome, SARS-CoV-2 susceptibility and severity, and host functions is complex and still not fully understood. Al Bataineh et al. evaluated the 16S rRNA gene-based microbial profiling in 143 subjects. The authors observed structural and compositional alterations in the gut microbiota of the SARS-CoV-2-infected group in comparison to non-infected controls and predicted the enrichment of several metabolic pathways, such as fatty acids, glycerolipid, glycerophospholipid, and amino acids metabolism induced by SARS-CoV-2 infection. The findings provide a new insight to enrich the understanding of SARS-CoV-2-related changes in gut microbiota, their metabolic capabilities, and potential screening biomarkers linked to disease severity.

Wu et al. investigated the relationship between SARS-CoV-2 infection and intestinal damage, specifically intestinal thrombosis. Different pathways related to the intestinal damage induced by the virus are presented and discussed. The intestinal thrombosis has a relevant impact on the gut damage associated with this infection. In severe cases of COVID-19, intestinal damage induced by SARS-CoV-2 can contribute to systemic inflammation and, consequently, to the dysfunction of other tissues or organs. Thus, the study highlights the complex relationship between SARS-CoV-2 and gut damage and points out to the importance of understanding the mechanisms involved in this process, aiming to address further studies to prevent intestinal damage in COVID-19 patients.

In the article by Cenci et al., the authors evaluated the potential effects of ozone therapy to treat COVID-19 patients, since this therapy has been suggested to have immunomodulatory effects during viral infections. Ozone therapy presented immunomodulatory effects during viral infection associated with modulation of oxidative stress by different mechanisms. Blood circulation and oxygen supply to tissues were also improved by the therapy, helping to reduce tissue damage caused by COVID-19. Thus, the study highlights the potential therapeutic effects of ozone therapy for COVID-19 treatment.

Hu et al. used simian immunodeficiency virus (SIV) infected non-human primate, a macaque model of human immunodeficiency virus (HIV) infection, to investigate the effect of this infection on mucosal susceptibility to SARS-CoV-2. The authors of this study focused on the expression of SARS-CoV-2 receptor/co-receptors and showed, in the gut mucosa, an early target organ of HIV, increased expression of angiotensin converting enzyme 2 (ACE2), a critical receptor for SARS-CoV-2 infection and dissemination. The authors also reported changes in the gut luminal content, which may be relevant for susceptibility to SARS-CoV-2, posing a potential threat for increased severity of the disease outcomes.

Dos Santos et al. presented and discussed how SARS-CoV-2 modulates metabolic processes in several tissues, including the central nervous system, skeletal muscle, kidneys, heart, liver, and intestine. They discussed how SARS-CoV-2 infection impairs metabolic pathways, resulting in oxidative stress, inflammation, and tissue damage and dysfunction. The authors also highlighted that it is important to comprehend the metabolic effects of the SARS-CoV-2 infection, in order to identify potential metabolic biomarkers and to develop therapeutic interventions targeting specific metabolic pathways to treat COVID-19 patients.

Lobato et al. indicated that the chronic inflammation and hyperglycemia, observed in obese and diabetic patients, may contribute to metabolic disturbances in leukocytes, thus negatively impacting on the response to SARS-CoV-2 infection. Obesity and T2D can favor the occurrence of the cytokine storm, implicated in the severity and high mortality risk of COVID-19 patients. Studies on this research field are still limited and further studies are crucial for the complete comprehension of the mechanisms involved in this process and for addressing of potential interventions to manage severe symptoms and complications in obese and T2D COVID-19 patients.

It has been demonstrated that SARS-CoV-2 infection increases reactive oxygen species (ROS) production and downregulates antioxidant defenses in host cells. Gain et al. reviewed the role played by oxidative stress on several aspects of the disease. They explored the correlation between oxidative stress and ACE2/Angiotensin II imbalance, discussing how it affects the expression of key membrane receptors and the organization of the plasma membrane, facilitating the virus entry in mammalian cells. They also presented evidence on how the modulation of redox-sensitive pathways (e.g., UPR, MAPK, Keap1-Nrf2-ARE, and NF-kB) could increase viral replication, inflammation, cytokine production, and apoptosis, leading to organ damage and contributing to acute symptoms and Post-Acute Sequelae of SARS-CoV-2. Finally, the paper summarized the current evidence on the benefits of antioxidant treatment for coronavirus infection.

## Author contributions

All authors prepared the first draft, critically reviewed, and edited the manuscript. All authors have read, reviewed, and approved of the final manuscript.
